# TMS-evoked potential in the dorsolateral prefrontal cortex to assess the severity of depression disease: a TMS-EEG study

**DOI:** 10.3389/fphar.2023.1207020

**Published:** 2023-06-05

**Authors:** Xingxing Li, Meng Chen, Qinqin Liu, Chao Zheng, Chang Yu, Guangwei Hou, Zan Chen, Yiqing Chen, Yinping Chen, Guidong Zhu, Dongsheng Zhou, Weiqian Xu

**Affiliations:** ^1^ Ningbo Kangning Hospital, Ningbo, Zhejiang, China; ^2^ Qingdao Mental Health Center, Qingdao, Shandong, China; ^3^ Yu Yao Third People’s Hospital, Ningbo, Zhejiang, China; ^4^ The Second People’s Hospital of Lishui, Lishui, Zhejiang, China; ^5^ Taizhou Second People’s Hospital, Taizhou, Zhejiang, China

**Keywords:** tmseeg, depression, psychology, DLPFC (dorsolateral prefrontal cortex), TEPS, HAMD, hamilton depression rating scale

## Abstract

**Objective:** The combined use of transcranial magnetic stimulation and electroencephalography (TMS-EEG), as a powerful technique that can non-invasively probe the state of the brain, can be used as a method to study neurophysiological markers in the field of psychiatric disorders and discover potential diagnostic predictors. This study used TMS-evoked potentials (TEPs) to study the cortical activity of patients with major depressive disorder depression (MDD) and the correlation with clinical symptoms to provide an electrophysiological basis for the clinical diagnosis.

**Methods:** A total of 41 patients and 42 healthy controls were recruited to study. Using TMS-EEG techniques to measure the left dorsolateral prefrontal cortex (DLPFC) ‘s TEP index and evaluate the clinical symptoms of MDD patients using the Hamilton Depression Scale-24 (HAMD-24).

**Results:** MDD subjects performing TMS-EEG on the DLPFC showed lower cortical excitability P60 index levels than healthy controls. Further analysis revealed that the degree of P60 excitability within the DLPFC of MDD patients was significantly negatively correlated with the severity of depression.

**Conclusion:** The low levels of P60 exhibited in DLPFC reflect low excitability in MDD; the P60 component can be used as a biomarker for MDD in clinical assessment tools.

## 1 Introduction

Depression is a mood disorder characterized by persistent depression and varying degrees of cognitive and behavioral changes (McCarron et al., 2021). Its high prevalence, high recurrence rate, high suicide rate, low recognition rate, and low cure rate have caused a severe burden to the patients themselves, their families, and society (Touloumis, 2021). Seeking potential biomarkers of depression patients can provide new strategies for early recognition and intervention of depression.

The dorsolateral prefrontal cortex (DLPFC) is considered the control center of emotion and cognition (Segal and Elkana, 2023). Patients with depression are believed to have functional and structural abnormalities in DLPFC, such as reduced volume, abnormal activity patterns, or abnormal available connection networks (Koenigs and Grafman, 2009). Repetitive transcranial magnetic stimulation (rTMS) is widely used to treat depression. The stimulation site is also selected for the left DLPFC, which is believed to effectively improve patients' clinical symptoms with depression (Kan et al., 2023). In most depression patients, the left DLPFC has shown low activity and low metabolism in functional neuroimaging studies, leading to the disturbance of neurotransmitter levels, such as glutamate and γ- Aminobutyric acid (Duman et al., 2019). Therefore, studying the EEG signals of left DLPFC has important significance for depression.

TMS generates a transient time-varying magnetic field, producing a transient electric field in the brain through electromagnetic conduction to depolarize or polarize neurons (Klomjai et al., 2015). TMS-EEG, which is formed by the combination of synchronous TMS and electroencephalography (EEG), has become a powerful tool for the non-invasive detection of human brain circuits, thus evaluating several cortical characteristics such as excitability and connectivity (Cao et al., 2021). Sun used TMS-EEG to assess whether baseline cortical inhibition in depression patients can predict the effect of treatment on suicidal ideation. Voneskos used TMS-EEG to compare the difference between DLPFC inhibition and excitation in depression and healthy controls (Sun et al., 2018; Voineskos et al., 2019). This technology has been applied to different clinical populations in recent years (C. T. Li et al., 2022; Strafella et al., 2023). Disease-related predictors can be found through this technology, providing markers for the pathophysiology of brain diseases.

As far as we know, few previous studies have used TMS-EEG to study depression, and the sample size is small. We want to find TEP in DLPFC induced by TMS-EEG as a potential biomarker to distinguish depression from healthy controls, and further clarify the relationship between this marker and the severity of depression, providing the theoretical basis for the clinical diagnosis and treatment of depression.

## 2 Materials and methods

### 2.1 Subject and assessment

The project recruited a total of 41 depression patients (9 male/32 female) in Ningbo Kangning Hospital from November 2021 to October 2022. Two psychiatrists evaluated all patients as assessed by the Structured Clinical Interview for the Diagnostic and Statistical Manual of Mental Disorders (DSM-V). Additional inclusion criteria were: 1) Age 16–65 years, 2) Hamilton Depression Scale-24 score (HAMD-24) ≥ 20 points; 3) All patients or guardians know the purpose of the experiment and sign an informed consent form.

Exclusion criteria: 1) Patients with severe somatic diseases, infectious diseases, and immune system diseases; 2) Accompany with other mental illnesses or severe neurological diseases.

**FIGURE 1:**
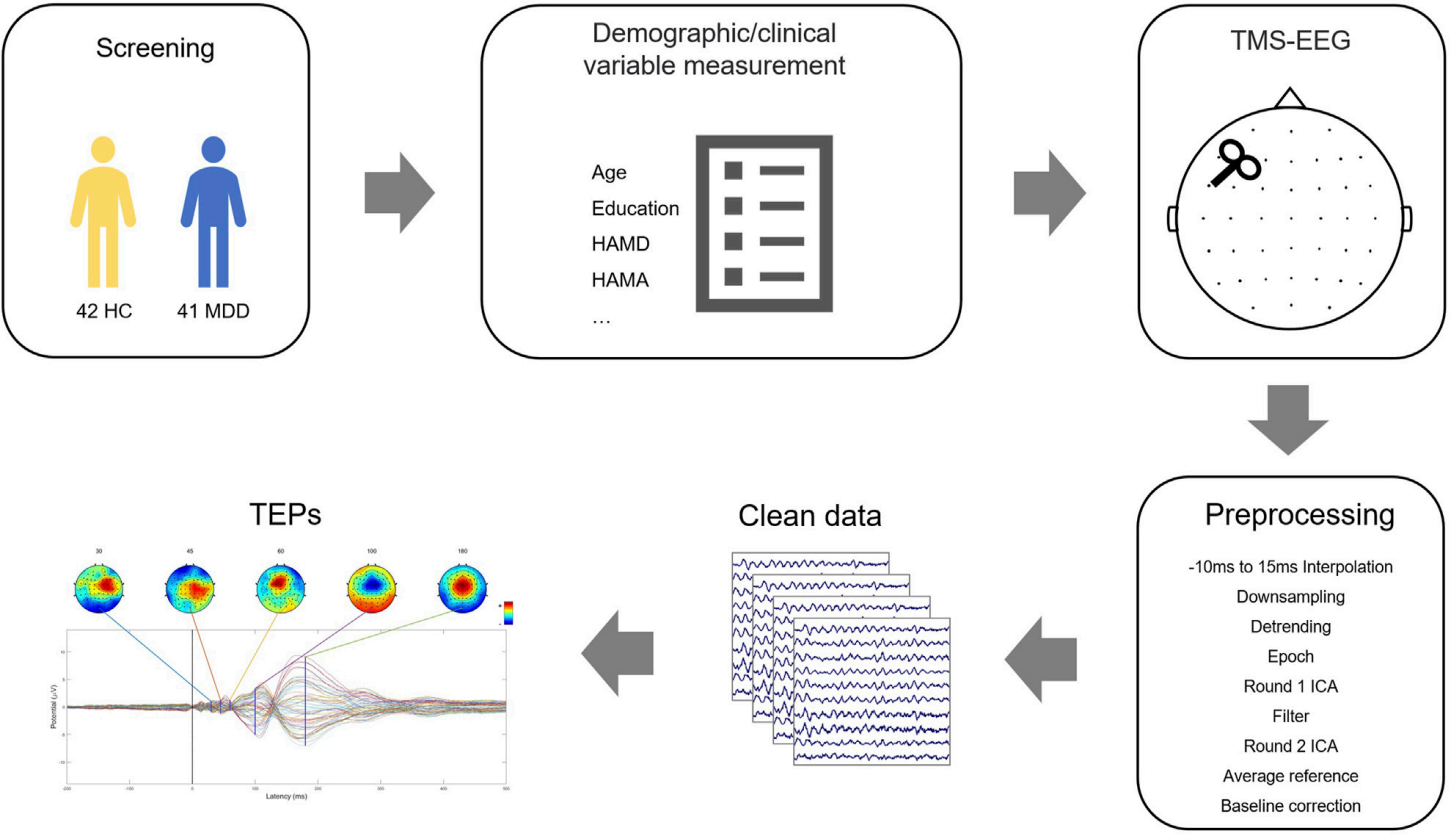
Schematic diagram of the experimental process and TMS-EEG data processing framework.

At the same time, we recruited 42 healthy controls in the community and matched them with MDD patients in terms of gender and age. We also excluded people with other mental diseases, basic diseases (such as stroke, diabetes, etc.), or alcohol disorders. The flow is shown in Figure 1.

The ethics committee of Ningbo Kangning Hospital approved the project, and all experimental procedures were conducted in accordance with the guidelines for human medical research (Helsinki Declaration). Before enrollment, the research protocol was registered at Chictr.org.cn (ChiCTR2100052007).

### 2.2 Assessment

The HAMD-24 items evaluated the depressive symptoms. The participants participated in the evaluation training of the scale before the study and re-evaluated each month to ensure that their correlation coefficient (ICC) remained above 0.8 to keep their scores consistent and reliable. The scores of HAMD-24 ≥ 20 points are classified as a depression group. The HAMD-24 scale can better reflect the severity of the condition. The more severe the condition, the higher the total score. Demographic information is a form of information collected through self-designed demographic statistics. Structured clinical interviews are used to process medical history.

### 2.3 TMS-EEG testing

TMS-EEG research collects data from TMS (Magstim Ltd, Oxford, United Kingdom) and TMS-compatible 64-channel EEG (Easycap, Germany). The figure-of-eight coil (Coil—D70-air film coil, Magstim) was placed over the F3 (roughly used to represent the left DLPFC brain region). The splay lock is tangent to the scalp and the handle points rearward at a 45° angle. Before TMS-EEG recording, all patients underwent a resting motion threshold (RMT) test to determine the stimulation intensity. RMT uses the necessary minimum stimulus intensity to determine the apparent motor response of the right abductor pollicis brevis (APB). Generally, at least five of the ten stimuli have an amplitude of ≥ 50 mV.

FCz and AFz are used as recording reference and ground electrodes, respectively. Use BrainVision Recorder software (BrainProducts, Germany) to record the EEG signal at a sampling rate of 25 kHz during the acquisition process. During the experiment, ensure the electrode impedance is kept below 5 k Ω. During TMS-EEG recording, 100 single TMS pulse stimuli were performed at F3 using a 100% RMT intensity. The interval between the individual stimuli was randomly generated over 5 s, and the patient kept their eyes open during the recording period. We also put earplugs on patients to prevent related auditory evoked potentials from being generated during stimulation.

### 2.4 TMS-EEG analysis

TMS-EEG data were analyzed offline using Matlab R2016b (MathWorks, United States) and in combination with the EEGLAB (Delorme and Makeig, 2004) and ARTIST (Wu et al., 2018) toolboxes. The preprocessing code was adapted from the fully automated artifact suppression algorithm for monopulse TMS-EEG (spTMS-EG), which consists of the following steps. The time window containing TMS pulse artifacts was first removed and replaced with an interpolation (−5–15 ms). Then the EEG data were downsampled to 1 kHz to reduce the file size. After data segmentation of TMS pulses (−2000–2000 ms), large fading artifacts are automatically removed using the first ICA based on thresholding. Band-pass filtering (1–100 Hz) is performed for slow drift and high-frequency noise. 50 Hz Alternating Current line noise artifacts are removed by a trap filter (48–52 Hz). Bad trials with signal amplitudes exceeding 3 standard deviations from the test mean are rejected. Bad channels were identified and interpolated by adjacent trials. Remaining artifacts, such as scalp muscle artifacts and eye artifacts, were automatically removed using a second ICA. Clean EEG data were then re-referenced to the common mean and corrected to the pre-TMS pulse baseline (−550 ms–−50 ms).

### 2.5 Statistical analysis

All of the data analyses used the Statistical Package for the Social Sciences (SPSS version 23, IBM). All data are expressed as mean ± SD; The data were tested for normality using Kolmogorov–Smirnov. For continuous variables, two-sample t-tests were used when comparing baseline (i.e., HC vs. MDD), but the Wilcoxon signed-rank test was used if the normality assumption was not satisfied. For categorical variables, chi-square tests were used. Analysis of covariance was used where necessary to control for the effect of covariates on the results.

In MDD patients, the Pearson correlation was used to analyze the correlation between the TEP index and the severity of depression. In addition, a linear logistic regression analysis was conducted to determine the factors related to depression. The statistically significant difference was set to *p* < 0.05; all tests were two-tailed.

## 3 Results

### 3.1 Demographic and clinical assessments

The demographic and clinical information of the participants is listed in Table 1. There was no significant difference between the MDD and HC groups in age and gender (all *p* > 0.05) except educational attainment (*p* < 0.001). Moreover, MDD exhibited higher depression and anxiety scores than healthy controls (all *p* < 0.001).

**TABLE 1:** Demographic and clinical information of the HC and MDD group.

		HC^a^ (*n* = 42)	MDD^b^ (*n* = 41)	*t (χ2)*	*p*
		*M(SE)*	*M(SE)*		
Gender				0.21	0.65
male		11	9		
female		31	32		
Age		31.48 (1.35)	29.29 (2.51)	0.77	0.44
Education (years)		15.67 (0.53)	10.95 (0.51)	6.42	<0.001***
HAMD-24		5.02 (0.31)	24.49 (0.10)	−20.22	<0.001***
HAMA-14		4.02 (0.30)	17.27 (1.02)	−12.46	<0.001***

^a^ *HC*, healthy controls.

^b^ *MDD*, individuals with major depressive disorder.

HDRS-24, Hamilton Depression Rating Scale-24; HAMA-14, Hamilton Anxiety Scale-14.

### 3.2 TEP differences between MDD and HC control in DLPFC

**FIGURE 2:**
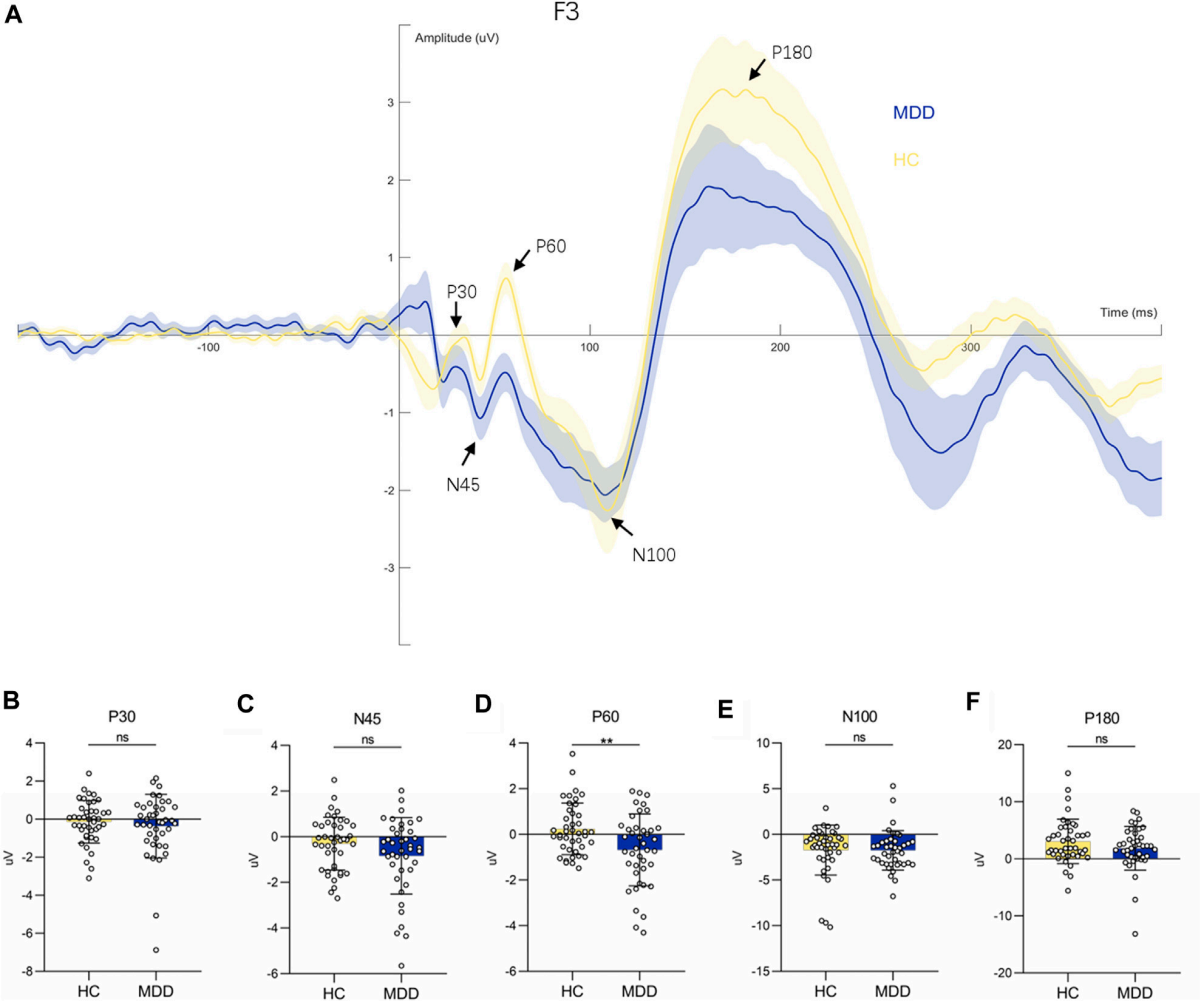
Waveform diagram at F3 electrode and comparison of each component. **(A)**: TEPs induced by stimulation of F3 electrodes. **(B–F)**: Comparison of P30, N45, P60, N100 and P180 components between HC and MDD respectively.

We investigated the potentials evoked by TMS pulses at the stimulation target, i.e., F3 (see Figure 2A). The P60 amplitude in individuals with MDD was significantly lower than that of the healthy controls (*F* = 7.44, *p* = 0.008**. see Figure 2D). The amplitudes of the other components did not reach significant differences between the two groups (for P30 amplitude, *F* = 0.40, *p* = 0.53; for N45 amplitude, *F* = 1.91, *p* = 0.17; for N100 amplitude, *F* = 0.05, *p* = 0.83; for P180 amplitude, *F* = 0.66, *p* = 0.42) see Figures 2B, C, E, F. Covariates in the RM MANOVA analysis included educational attainment.

### 3.3 Association between depressive symptoms and P60

**FIGURE 3:**
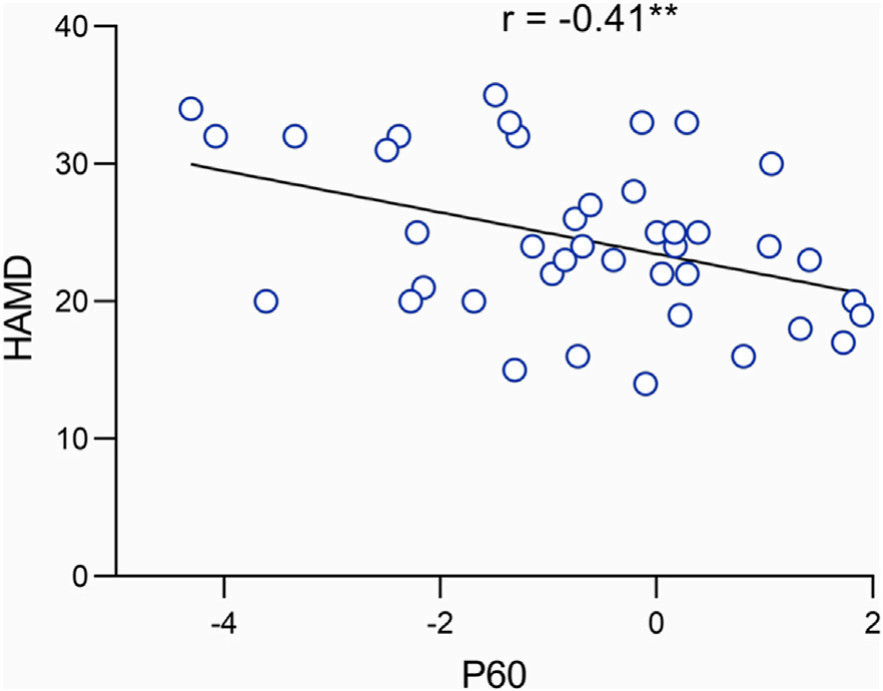
Correlation of P60 component with depression severity.

We performed a Pearson correlation analysis to investigate further the role of P60 abnormalities in the neuropathology of MDD. The results indicated that the amplitude of the P60 component was significantly and negatively correlated with the severity of depression (*r* = −0.41, *p* = 0.008**. See Figure 3).

### 3.4 Regression analysis

To investigate whether P60 could be used as a biomarker to examine depressive symptoms, we created a regression model of depression severity. Considering the mismatch in education attainment between the individuals with MDD and HC, it was necessary to control for educational attainment as a covariate. The results indicated that the P60 could significantly predict depressive symptoms (*B* = −1.5, CI -2.61—−0.40, *t* = −2.75, *p* = 0.009**).

## 4 Discussion

Compared to previous studies using TMS-EEG techniques to assess cortical inhibition. This study provides evidence for abnormal DLPFC excitability in MDD patients compared with healthy control. The results showed that the cortical excitability index (P60) measured by TMS-EEG was lower in MDD. More importantly, this decreased cortical excitability in DLPFC correlates with the severity of depressive symptoms. Taken together, the P60 index induced by TMS may be of great significance for the diagnosis of depression.

Abnormal DLPFC excitability is an important neural mechanism of depression. Previous experiments have reportedly typically used TMS-evoked motor potentials (MEPs) to study abnormal cortical excitability and inhibition in MDD patients (Oliveira-Maia et al., 2017; Khedr et al., 2020; Castricum et al., 2022; Li et al., 2023). However. the non-motor cortex has been less studied (mostly using TMS-evoked potentials (TEPs), and previous studies have mostly been based on global mean field amplitudes used to reveal changes in cortical excitability at the whole brain level (Cao et al., 2021). Furthermore, TEPs are more sensitive than MEPs in studying cortical excitability. this can be recorded in local and distal electrodes, allowing the study of the spread of activation in cortical areas. And TEPs are evoked only after stimulation of intact functional areas of the cortex, providing direct evidence that TEPs reflect cortical activity rather than electrical or physiological artifacts (Jannati et al., 2023). The DLPFC is particularly important in the neuropathology of MDD, and TMS-EEG allows direct assessment of its excitatory and inhibitory properties, with hypoexcitability of the DLPFC associated with impaired emotion regulation and cognitive control, thereby increasing the risk of undesirable behaviors in response to negative stimuli (Koenigs and Grafman, 2009). Previous studies have identified abnormal expression of DLPFC in MDD patients, mainly in the form of low activity and hypometabolism of DLPFC in neuroimaging studies (Ma, 2015; Zhang et al., 2021). Moreover, improved clinical symptoms in depressed patients after treatment with antidepressants are associated with increased activity of the left DLPFC (George and Post, 2011). Our findings are consistent with the above conclusion that depressed patients with DLPFC exhibit hypoactivation, i.e., low amplitude of p60, which is thought to correlate with excitability and inhibition.

Some evidence suggests that the balance of inhibition and excitation in the DLPFC is altered in patients with major depression. Animal models of major depression show a decrease in GABAergic interneurons, mainly in the DLPFC region, while human imaging studies using magnetic resonance spectroscopy have also shown altered GABA levels in the DLPFC (Rajkowska et al., 2007; Godfrey et al., 2018). These studies support that prefrontal inhibition is an important feature of major depression, but little has been done to link altered prefrontal excitability to the neuropathological mechanisms of MDD. Previous studies have shown that P60 reflects cortical excitatory mechanisms involving NMDA and glutamatergic-mediated neurotransmission processes (Gordon et al., 2023). Noda et al. found a correlation between P60 and N100 in healthy individuals induced using a short-interval intracortical inhibition paradigm. This correlation was suggested to be explained by a correlation with glutamate and GABA receptor-mediated excitation-inhibition balance (Cash et al., 2017). Belardinelli et al. investigated the effects of anti-glutamatergic drugs on TMS-evoked potentials and showed that the effects of AMPA receptor antagonists on TMS-evoked potentials reduced the amplitude of P60, reflecting that P60 may be associated with AMPA receptor activation-mediated glutamatergic signal propagation. It is further suggested that P60 may reflect glutamatergic-mediated cortical excitability (Belardinelli et al., 2021). Glutamate is a central factor in mood disorders. It has been found that the excitatory neurotransmitter glutamate levels are generally reduced in MDD, especially in the prefrontal cortex (Abdallah et al., 2014; Henter et al., 2021; Kantrowitz et al., 2021). Also, glutamate receptor antagonists are more effective in combination with transcranial magnetic therapy for MDD (Best et al., 2019; Elkrief et al., 2022). In the present study, we found that the amplitude of P60 in depressed patients was positively correlated with the severity of depressive symptoms. This suggests a possible significant intrinsic association of P60 with clinical depressive symptoms. This study suggests that there may be a significant intrinsic association between prefrontal cortex excitability and clinical depressive symptoms. And based on previous studies, this cortical excitability is associated with glutamatergic activity. Therefore, we hypothesize that restoring glutamate-related cortical excitability in DLPFC may be one of the potential neural mechanisms for treating depression, and further research is necessary to verify this in the future. The above findings may help clinicians objectively measure patients' conditions, facilitate the implementation of individualized treatment plans and improve the treatment success rate.

There are several limitations to this study. Firstly, the small sample size makes our sample more susceptible to random factors. Therefore, increasing the sample size would have produced more stable results. Secondly, it can be due to the nature of the cross-sectional study design that the present study could not confirm whether P60 amplitude recovers with symptom improvement, and furthermore, a causal relationship between DLPFC excitability and depression severity could not be obtained. Future longitudinal studies are needed to explore this aspect more. Thirdly, each patient was taking antidepressants during the clinical trial and we cannot exclude that medication may have an effect on TEPs, which is not conducive to exploring the true predictive effect of P60. In the future, further studies are needed to verify whether group differences in P60 persist in the absence of antidepressants. In addition, this study used the F3 electrode point location of the EEG cap to refer to the DLPFC, which was not accurate for every participant. Therefore, it is necessary to use MRI-based neuronavigation systems to identify precise targets in future studies.

Finally, the study lacked serum or cerebrospinal fluid examinations to identify potential molecular biomarkers (such as BDNF levels) and to explain the relationship between neurotransmitters, and the TMS-EEG indicators may have new findings.

In summary, this report applied TMS-EEG to confirm that prefrontal cortex excitability is decreased in patients with major depressive disorder and that there is a correlation between this diminished DLPFC excitability and the severity of depressive symptoms. TMS-EEG objectively and directly assesses the excitatory properties of the cortex without relying on the subjective involvement of the participant and has excellent retest reliability. This study provides evidence for an association between P60 and depression severity, which is expected to be integrated into clinical assessment tools in the future.

## Data Availability

The raw data supporting the conclusion of this article will be made available by the authors, without undue reservation.
